# Prognostic value of National Early Warning Score and Modified Early Warning Score on intensive care unit readmission and mortality: A prospective observational study

**DOI:** 10.3389/fmed.2022.938005

**Published:** 2022-08-04

**Authors:** Ata Mahmoodpoor, Sarvin Sanaie, Seied Hadi Saghaleini, Zohreh Ostadi, Mohammad-Salar Hosseini, Naeeme Sheshgelani, Amir Vahedian-Azimi, Abbas Samim, Farshid Rahimi-Bashar

**Affiliations:** ^1^Research Center for Integrative Medicine in Aging, Aging Research Institute, Tabriz University of Medical Sciences, Tabriz, Iran; ^2^Department of Anesthesiology and Intensive Care, Faculty of Medicine, Tabriz University of Medical Sciences, Tabriz, Iran; ^3^Student Research Committee, Tabriz University of Medical Sciences, Tabriz, Iran; ^4^Trauma Research Center, Nursing Faculty, Baqiyatallah University of Medical Sciences, Tehran, Iran; ^5^Chemical Injuries Research Center, Systems Biology and Poisonings Institute, Baqiyatallah University of Medical Sciences, Tehran, Iran; ^6^Anesthesia and Critical Care Department, Hamadan University of Medical Sciences, Hamadan, Iran

**Keywords:** intensive care unit, National Early Warning Score, Modified Early Warning Score, readmission, mortality, prognosis

## Abstract

**Background:**

Modified Early Warning Score (MEWS) and National Early Warning Score (NEWS) are widely used in predicting the mortality and intensive care unit (ICU) admission of critically ill patients. This study was conducted to evaluate and compare the prognostic value of NEWS and MEWS for predicting ICU readmission, mortality, and related outcomes in critically ill patients at the time of ICU discharge.

**Methods:**

This multicenter, prospective, observational study was conducted over a year, from April 2019 to March 2020, in the general ICUs of two university-affiliated hospitals in Northwest Iran. MEWS and NEWS were compared based on the patients’ outcomes (including mortality, ICU readmission, time to readmission, discharge type, mechanical ventilation (MV), MV duration, and multiple organ failure after readmission) using the univariable and multivariable binary logistic regression. The receiver operating characteristic (ROC) curve was used to determine the outcome predictability of MEWS and NEWS.

**Results:**

A total of 410 ICU patients were enrolled in this study. According to multivariable logistic regression analysis, both MEWS and NEWS were predictors of ICU readmission, time to readmission, MV status after readmission, MV duration, and multiple organ failure after readmission. The area under the ROC curve (AUC) for predicting mortality was 0.91 (95% CI = 0.88–0.94, *P* < 0.0001) for the NEWS and 0.88 (95% CI = 0.84–0.91, *P* < 0.0001) for the MEWS. There was no significant difference between the AUC of the NEWS and the MEWS for predicting mortality (*P* = 0.082). However, for ICU readmission (0.84 vs. 0.71), time to readmission (0.82 vs. 0.67), MV after readmission (0.83 vs. 0.72), MV duration (0.81 vs. 0.67), and multiple organ failure (0.833 vs. 0.710), the AUCs of MEWS were significantly greater (*P* < 0.001).

**Conclusion:**

National Early Warning Score and MEWS values of >4 demonstrated high sensitivity and specificity in identifying the risk of mortality for the patients’ discharge from ICU. However, we found that the MEWS showed superiority over the NEWS score in predicting other outcomes. Eventually, MEWS could be considered an efficient prediction score for morbidity and mortality of critically ill patients.

## Introduction

Readmission to the intensive care units (ICUs) is associated with poor patient outcomes, including higher mortality, a longer length of stay, and higher adverse event rates (1–3). In addition, ICU readmissions bring financial burden and wastefulness to the patient flow of the healthcare system (4, 5). Readmitted patients reduce ICU bed availability and, probably, the efficiency of the ICU facilities (6, 7). The intensivist usually decides to discharge patients from the ICU based on clinical evaluations (8, 9). However, several other non-clinical factors contribute to such decisions – including the high demand and need for ICU beds by emergency and surgical departments – making the discharge decision a complex, challenging, and risky care transfer process (10, 11). These factors may lead to an early and inadequate discharge of patients, which increases the risk of readmission, as up to 42% of patients discharged early are eventually readmitted to the ICU (12). Hence, several attempts have been made to optimize and prioritize ICU discharges, either by identifying risk factors associated with ICU readmission (9, 13) or developing readmission prediction models (14, 15). These models for mortality and readmission after ICU discharge have shown diverse accuracy. Although prospective validation is warranted for these scoring systems, they speculate that these models could be valuable assistance to clinicians for ICU discharge planning.

Several Early Warning Scores (EWSs) with different designs have been developed to diagnose early signs of deterioration in a patient’s conditions and initiate further medical care and possible ICU admission (16–18). Since a critical state usually follows specific deteriorations in the patient’s physiological signs, monitoring these signs could help the physicians predict the patient’s outcomes (19–21). One of the common EWSs is the Modified Early Warning Score (MEWS), validated in 2001 in the United Kingdom as a bedside tool to identify patients at risk for catastrophic events, including death or readmission to ICU (22). National Early Warning Score (NEWS) is another EWS introduced in 2012 by the Royal College of Physicians (23). The NEWS score identifies the patients at risk of deterioration and facilitates prompt critical care intervention. Also, many studies have shown the capability of NEWS in predicting the degree of illness (18, 24). Several studies have explored the association between these risk scores and hospital admission. The findings suggest that these risk scores could also be used as triage tools to identify patients requiring hospital admission (22, 25, 26).

Due to the lack of studies comparing NEWS and MEWS risk-scoring systems in ICU settings, it is still unclear which risk-scoring system is superior as a triage tool for ICU readmission and predicting mortality of critically ill patients. Considering the lack of information and the inconsistency in the cut-off values, this study was conducted to evaluate and compare the prognostic value of NEWS and MEWS for predicting ICU readmission, mortality, and related outcomes in critically ill patients at the time of ICU discharge.

## Materials and methods

### Study design and population

This multicenter, prospective, observational study was conducted over a year, from April 2019 to March 2020, in the general intensive care units (ICUs) of two university-affiliated hospitals in Northwest Iran, to evaluate and compare the prognostic value of NEWS and MEWS scores for predicting ICU readmission, mortality and related outcomes in critically ill patients at the time of discharge from the ICU. All adult (over 18 years old) patients alive at the time of ICU discharge were eligible to enroll in this study, regardless of the medical diagnoses and underlying comorbidities. However, patients were excluded if they were: (a) stayed in the ICU for less than 48 h (such as postoperative patients), (b) patients directly discharged home or transferred to other medical centers, (c) patients discharged for palliative care, and (d) patients readmitted to the ICU for the second time. Patients who no longer needed mechanical ventilation (MV), vasopressor support, and renal replacement therapies were discharged from the ICU with appropriate levels of consciousness and transferred to general wards. Subsequently, all patients were followed up for 2 weeks to identify readmitted patients.

### Ethical considerations

The protocol study was reviewed and approved by the Research Ethics Committees of Islamic Azad University-Tabriz Branch (IR.TBZMED.REC.1397.994), following the Declaration of Helsinki of the World Medical Association (27). Written informed consent was obtained from the patients or their legally accepted representatives. In addition, the study was conducted and reported in accordance with the recommendations of the Strengthening the Reporting of Observational Studies in Epidemiology (STROBE) statement (28).

### Data collection

Demographic characteristics and clinical data, including comorbidities, reasons for admission (medical, surgical, or emergency), the severity of illness [based on Acute Physiology and Chronic Health Evaluation IV (APACHE-IV) and Sequential Organ Failure Assessment (SOFA) scores], consciousness state, and vital signs (respiratory rate, peripheral oxygen saturation (SpO_2_), systolic/diastolic blood pressure, heart rate, pulse rate, and body temperature) upon ICU admission were recorded for all patients. Additionally, we collected the information, including the status and type of multiple organ failure, mechanical ventilation (MV) status, MV duration, length of stay (LOS) in the ICU, and the NEWS and MEWS scores at the time of ICU discharge. All data were collected and analyzed by researchers completely independent of the clinical decision-makers.

### Calculation of National Early Warning Score and Modified Early Warning Score scores

A trained nurse calculated the NEWS and MEWS scores for all patients who were alive at the time of ICU discharge using physiological parameters. NEWS scores were obtained by nursing staff at the ICUs, including the following seven common vital signs parameters: Respiratory rate (RR), peripheral oxygen saturation (SpO_2_) measured by pulse oximetry, supplementary oxygen, systolic arterial blood pressure (SBP), pulse rate (PR), body temperature (T), and AVPU (Alert, responds to Voice, responds to Pain, Unresponsive) score based on the Glasgow Coma Scale (GCS) [The AVPU score was derived from the GCS as follows: *A* = 14–15, *V* = 9–13, *P* = 4–8, *U* = 3] (29). Patients with a score between 0 and 4 are considered low risk, those with a score of 5 or 6 are considered medium risk, and patients with a score ≥ 7 are considered high risk (29). The MEWS consists of five physiological variables, including systolic blood pressure (SBP), heart rate (HR), respiratory rate (RR), body temperature (T), and AVPU score based on the GCS. Determining a MEWS score involves assigning a number between 0 and 3 to each of the six vital signs. Patients with scores between 2 and 4 are considered at medium risk and should remain under specialized care and be assessed again in 2 to 8 h. Those with a score ≥ 5 are considered at high risk for mortality and being moved to ICU (22).

### Outcomes

The primary outcomes were mortality and readmission to the ICU. The secondary outcomes were the type of discharge from ICU and subgroups of consequences related to the readmission, such as mechanical ventilation, duration of mechanical ventilation, and multiple organ failure.

### Statistical analysis

Data were expressed as mean ± standard division (SD) or median with interquartile range (IQR) for continuous variables, and frequencies with percentages (%) for categorical characteristics. The Shapiro-Wilk test was used to determine whether data were normally distributed. To compare the NEWS and MEWS scores according to the outcomes and subgroups of outcomes, we used Mann–Whitney as a non-parametric test for non-normal distributions. Univariate and multivariate binary logistic regression analyses were performed to evaluate associations of NEWS and MEWS scores with the outcomes. Each variable was first tested by univariate analysis with odds ratios (OR) and 95% confidence intervals (95% CI). In multivariate analysis, based on conditional logistic regression, variables with a *p*-value < 0.05 in the univariate analyses were proposed for entry into the model. To assess the predictive prognostic efficacy of the NEWS and MEWS scores, we performed receiver operating characteristic (ROC) curves and calculated the area under the curves (AUC). AUC figures were calculated alongside sensitivity (SN), specificity (SP), positive likelihood ratio (LR +), negative likelihood ratio (LR-), positive predictive value (PPV), negative predictive value (NPV), and Youden index to find appropriate cut-offs. In addition, we compared the ROC of NEWS and MEWS scores using the DeLong test. According to the general guide, AUC between (0.9-1.0), (0.8-0.9), (0.7-0.8), and (0.6-0.7) was considered as excellent, good, fair, and poor, respectively. Statistical analysis was performed using SPSS Statistics 21.0 (SPSS Inc., Chicago, IL, United States) and MedCalc.^1^ In all analyses, *p*-values less than 0.05 were considered significant.

## Results

### Characteristics of patients

In total, 410 patients were selected for this study. The basic information and clinical characteristics of the patient population are listed in Table 1. The median age (IQR) of the patients was 59 (49.75–69) years, and 223 (56.8%) patients were male. Nearly half of the patients (*n* = 185, 45.1%) had comorbidities, and 25 (6.1%) had more than two underlying diseases. The most common reason of admission was surgical (*n* = 272, 66.3%) followed by medical (*n* = 102, 24.9%) and emergency (*n* = 36, 8.8%). The median (IQR) of APACHE IV and SOFA scores of the patients were 23.5 (21–26) and 9 (6–13.25), respectively. More than half of patients had multiple organ failure (*n* = 286, 69.8%) and underwent MV (*n* = 273, 66.6%). The median (IQR) length of stay in the ICU and MV duration were 9 (6–13.25) and 8 (5–12) days, respectively.

**TABLE 1:** Demographic and clinical characteristics data of all patients (*n* = 410).

Variables		Frequency

Age (years)	Median (IQR)	59 (49.75–69)
Gender	Male (%)	223 (56.8)
	Female (%)	177 (43.2)
Comorbidities	Yes (only one disease,%)	160 (39)
	Yes (more than two diseases,%)	25 (6.1)
	No (%)	225 (54.9)
Types of comorbidities	CVA (%)	26 (6.3)
	Malignancy (%)	14 (3.4)
	IHD (%)	51 (12.4)
	HTN (%)	43 (10.5)
	DM (%)	32 (7.8)
	CHF (%)	33 (8)
	HLP (%)	7 (1.7)
	MI (%)	3 (0.7)
	ESRD (%)	1 (0.2)
Reasons of admission	Medical (%)	102 (24.9)
	Surgical (%)	272 (66.3)
	Emergency (%)	36 (8.8)
APACHE IV	Median (IQR)	23.5 (21–26)
SOFA	Median (IQR)	9 (6–13.25)
Multiple organ failure	Yes (%)	286 (69.8)
	No (%)	124 (30.2)
Type of multiple organ failure	Respiratory (%)	173 (42.2)
	Cardiovascular (%)	57 (13.9)
	Neurologic (%)	92 (22.4)
	Renal (%)	67 (16.3)
ICU length of stay (LOS)	Median (IQR)	9 (6–13.25)
Mechanical ventilation	Yes (%)	273 (66.6)
(MV)	No (%)	137 (33.4)
MV duration (days)	Median (IQR)	8 (5–12)
NEWS score	Median (IQR)	4 (3–4)
MEWS score	Median (IQR)	3 (3–3)

Cerebrovascular accident (CVA), Ischemic heart disease (IHD), Hypertension (HTN), Diabetes mellitus (DM), Congestive heart failure (CHF), Hyperlipidemia (HLP), Myocardial infarction (MI), End-stage renal disease (ESRD), Acute Physiology and Chronic Health Evaluation IV (APACHE-IV), Sequential Organ Failure Assessment (SOFA), National Early Warning Score (NEWS), Modified Early Warning Score (MEWS).

### Characteristics of readmitted patients

A total of 50 (12.2%) ICU patients discharged to the general ward were readmitted within 2 to 12 days, with a median (IQR) time of 4 (3–4) days. Clinical characteristics of readmitted patients and the main reasons for readmission are presented in Table 2. Of 50 patients readmitted to the ICU, 39 (78%) underwent MV. The median (IQR) MV duration in readmitted patients was 6 (5–6) days. Organ failure was present in 48 (96%) readmitted patients.

**TABLE 2:** Clinical characteristics of patients readmitted to the ICU (*n* = 50).

Variables		Frequency
Reasons of readmission	Embolism (%)	1 (2)
	Consciousness disorder (%)	9 (18)
	Cardiovascular (%)	6 (12)
	Renal (%)	4 (8)
	Brain (%)	3 (6)
	Pneumonia (%)	3 (6)
	Respiratory failure (%)	24 (48)
Time to readmission	Median (IQR) days	4 (3–6)
Re-mechanical ventilation	Yes (%)	39 (78)
	No (%)	11 (22)
MV duration readmission	Median (IQR) days	6 (5–6)
Multiple organ failure readmission	Yes (%)	48 (96)
	No (%)	2 (4)
Type of multiple organ failure	Respiratory (%)	20 (40)
	Cardiovascular (%)	9 (18)
	Neurologic (%)	12 (24)
	Renal (%)	9 (18)

### Comparison of National Early Warning Score and Modified Early Warning Score scores according to outcomes

Table 3 presents the detailed comparison of NEWS and MEWS scores among the patients regarding mortality, type of discharge, readmission, time to readmission, MV status, MV duration, and organ failure after readmission. Comparing NEWS and MEWS scores between outcomes showed statistically significant differences, as the median scores of NEWS and MEWS were significantly higher in non-survivors, readmitted patients, patients with lower (<4) days to readmission, those who underwent MV, patients with higher (≥6) days of MV, and patients with multiple organ failure. However, no significant differences were observed between median scores of NEWS (*p*-value = 0.332) and MEWS (*p*-value = 0.447) in the patients with planned and unplanned types of discharge.

**TABLE 3:** Comparison of NEWS and MEWS scores according to the outcomes and subgroups of outcomes.

Outcomes		Frequency (%)	NEWS score			MEWS score		
				
			Median (IQR)	Mean Rank	*P*-value	Median (IQR)	Mean Rank	*p-value*
Mortality (*n* = 410)	Yes	32 (7.8)	5 (4–6)	362.7	**<0.0001**	5 (4–5)	193.3	**<0.0001**
	No	378 (92.2)	4 (3–4)	192.2		3 (3–3)	349.5	
Discharge type of ICU (*n* = 410)	Planned	392 (95.6)	3 (3–4)	206.6	0.332	3 (3–3)	204.7	0.447
	Unplanned	18 (4.4)	4 (3–4)	181.5		3 (2–4)	222.9	
ICU Readmission	Yes	50 (12.2)	4 (4–4.25)	281.5	**<0.0001**	4 (4–4)	327.4	**<0.0001**
	No	360 (87.8)	3.5 (3–4)	194.9		3 (3–3)	188.5	
Time to readmission (*n* = 50)	≥4 days	37 (76)	4 (3–4)	198.9	**<0.0001**	3 (3–3)	193.4	**<0.0001**
	<4 day	13 (24)	4 (4–4)	271.2		4 (4–4)	327.2	
MVReadmission (*n* = 50)	Yes	39 (78)	4 (4–5)	287.1	**<0.0001**	4 (4–4)	327.4	**<0.0001**
	No	11 (22)	4 (3–4)	196.9		3 (3–3)	192.6	
MVreadmission duration (*n* = 50)	≥6 days	22 (44)	4 (4–5)	273.5	**0.002**	4 (3.7–4)	326.8	**<0.0001**
	<6 days	28 (56)	4 (3–4)	201.6		3 (3–3)	198.6	
Multiple organ failure readmission (*n* = 50)	Yes	48 (96)	4 (4–4.75)	281.6	**<0.0001**	4 (4–4)	326.2	**<0.0001**
	No	2 (4)	4 (3–4)	195.4		3 (3–3)	189.5	

*p-value* < 0.05 considered significant. The *p*-value was evaluated based on the Mann–Whitney test.

### Logistic regression findings

Tables 4, 5 present the univariable and multivariable binary logistic regression analyses to evaluate associations of NEWS and MEWS scores to predict outcomes. In univariable analysis, an increase in mortality risk was observed in a higher NEWS score (OR: 18.58, 95% CI: 8.45–40.86, *p*-value < 0.001) and MEWS score (OR: 12.19, 95% CI: 6.43–23.11, *p*-value < 0.001). However, multivariable analysis showed that the higher NEWS was only associated with mortality (OR: 6.51, 95% CI: 1.81–23.43, *p*-value = 0.004). In addition, the multivariable binary logistic regression model identified that the higher NEWS and MEWS scores upon discharge were associated with readmission, lower time to readmission, the risk of undergoing MV after readmission, higher MV duration, and the risk of multiple organ failure after readmission.

**TABLE 4:** Univariable and multivariable binary logistic regression analysis to evaluate associations of NEWS and MEWS scores to predict mortality, readmission, discharge type, and time to readmission.

Variables	Univariate		Multivariate	
		
	OR (95% CI)	*P-value*	OR (95% CI)	*P-value*
Mortality (Yes vs. No)				
Age	1.01 (0.97–1.03)	0.741	–	–
Gender (Male vs. Female)	1.17 (0.57–2.426)	0.66	–	–
Comorbidities (Yes vs. No)	2.90 (1.33–6.29)	0.007*	3.23 (0.82–12.6)	0.092
Comorbidities (≥2 vs. 1)	5.60 (2.14–14.66)	<0.001*	2.19 (0.37–12.8)	0.385
SOFA	2.05 (1.67–2.52)	<0.001*	0.64 (0.23–1.74)	0.385
APACHE IV	1.48 (1.32–1.65)	<0.001*	1.50 (0.87–2.58)	0.137
NEWS score	18.58 (8.45–40.86)	<0.001*	6.51 (1.81–23.4)	0.004*
MEWS score	12.19 (6.43–23.12)	<0.001*	2.62 (0.86–7.95)	0.089
ICU Readmission (Yes vs. No)				
Age	1.01 (0.98–1.03)	0.382	–	–
Gender (Male vs. Female)	2.17 (1.18–3.97)	0.012*	2.36 (1.18–4.70)	0.015*
Comorbidities (Yes vs. No)	1.50 (0.82–2.71)	0.18	–	–
Comorbidities (≥2 vs. 1)	1.88 (0.67–5.28)	0.225	–	–
SOFA	1.30 (1.13–1.49)	<0.001*	1.02 (0.54–1.93)	0.935
APACHE IV	1.14 (1.06–1.22)	<0.001*	0.95 (0.68–1.33)	0.798
NEWS score	2.17 (1.52–3.10)	<0.001*	2.24 (1.11–5.54)	<0.001*
MEWS score	3.43 (2.36–4.99)	<0.001*	8.12 (3.71–17.80)	<0.001*
Discharge type (Unplanned vs. Planned)				
Age	0.93 (0.91–0.97)	0.001*	0.93 (0.90–0.97)	0.002*
Gender (Male vs. Female)	1.05 (0.41–2.73)	0.911	–	–
Comorbidities (Yes vs. No)	0.76 (0.29–2.01)	0.588	–	–
Comorbidities (≥2 vs. 1)	0.87 (0.35–3.54)	0.998	–	–
SOFA	0.66 (0.49–0.88)	0.006*	0.67 (0.27–1.65)	0.392
APACHE IV	0.82 (0.71–0.96)	0.013*	1.03 (0.64–1.65)	0.884
NEWS score	0.67 (0.32–1.41)	0.299	–	–
MEWS score	1.16 (0.65–2.09)	0.604	–	–
Time to readmission (< 4 days vs. ≥4 days)				
Age	1.01 (0.97–1.03)	0.862	–	–
Gender (Male vs. Female)	1.62 (0.82–3.19)	0.164	–	–
Comorbidities (Yes vs. No)	1.03 (0.52–2.04)	0.916	–	–
Comorbidities (≥2 vs. 1)	0.87 (0.19–3.84)	0.854	–	–
SOFA	1.32 (1.14–1.54)	<0.001*	1.15 (0.58–2.27)	0.672
APACHE IV	1.15 (1.06–1.25)	<0.001*	0.93 (0.65–1.33)	0.709
NEWS score	1.87 (1.26–2.78)	0.002*	3.21 (1.08–6.51)	0.001*
MEWS score	2.84 (1.94–4.17)	<0.001*	7.04 (2.95–16.77)	<0.001*

*P-value < 0.05 considered significant, Abbreviations: Odds ratio (OR), Confidence Interval (CI), Acute Physiology and Chronic Health Evaluation IV (APACHE-IV), Sequential Organ Failure Assessment (SOFA), National Early Warning Score (NEWS), Modified Early Warning Score (MEWS).

**TABLE 5:** Univariable and multivariable binary logistic regression analysis to evaluate associations of NEWS and MEWS scores to predict MV status, MV duration, and organ failure after readmission.

Variables	Univariate		Multivariate	
		
	OR (95% CI)	*P-value*	OR (95% CI)	*P-value*
MV after Readmission (Yes vs. No)				
Age	1.01 (0.98–1.03)	0.493	–	–
Gender (Male vs. Female)	2.02 (1.03–3.96)	0.039	–	–
Comorbidities (Yes vs. No)	1.47 (0.76–2.85)	0.252	–	–
Comorbidities (≥2 vs. 1)	2.58 (0.91–7.31)	0.074	–	–
SOFA	1.32 (1.14–1.54)	<0.001*	0.98 (0.51–1.90)	0.967
APACHE IV	1.16 (1.07–1.25)	<0.001*	0.98 (0.69–1.38)	0.917
NEWS score	2.28 (1.55–3.35)	<0.001*	1.35 (1.10–4.78)	0.011*
MEWS score	3.15 (2.15–4.64)	<0.001*	5.38 (2.42–11.96)	<0.001*
MVduration after readmission (≥6 days vs. <6 days)				
Age	1.00 (0.97–1.04)	0.771	–	–
Gender (Male vs. Female)	2.98 (1.19–7.49)	0.020*	3.01 (1.14–7.95)	0.026*
Comorbidities (Yes vs. No)	1.23 (0.52–2.90)	0.637	–	–
Comorbidities (≥2 vs. 1)	2.62 (0.72–9.55)	0.143	–	–
SOFA	1.25 (1.03–1.51)	0.020*	0.67 (0.29–1.53)	0.350
APACHE IV	1.12 (1.02–1.24)	0.016*	1.25 (0.81–1.95)	0.313
NEWS score	1.90 (1.17–3.08)	0.009*	2.18 (1.10–4.78)	0.011*
MEWS score	2.92 (1.87–4.56)	<0.001*	12.39 (3.36–45.67)	<0.001*
Multiple organ failure after readmission (Yes vs. No)				
Age	1.01 (0.98–0.03)	0.485	–	–
Gender (Male vs. Female)	2.43 (1.31–4.53)	**0.0058**	2.67 (1.32–5.40)	0.006*
Comorbidities (Yes vs. No)	1.37 (0.75–2.51)	0.304	–	–
Comorbidities (≥2 vs. 1)	1.98 (0.71–5.57)	0.191	–	–
SOFA	1.29 (1.12–1.48)	<0.001*	1.09 (0.57–2.06)	0.784
APACHE IV	1.13 (1.05–1.22)	<0.001*	0.91 (0.65–1.27)	0.594
NEWS score	2.17 (1.51–3.11)	<0.001*	3.27 (1.12–8.59)	0.001*
MEWS score	3.33 (2.29–4.84)	<0.001*	7.44 (3.39–16.36)	<0.001*

*P-value < 0.05 considered significant. Odds ratio (OR), Confidence Interval (CI), Acute Physiology and Chronic Health Evaluation IV (APACHE-IV), Sequential Organ Failure Assessment (SOFA), National Early Warning Score (NEWS), Modified Early Warning Score (MEWS).

### Predicting outcomes by National Early Warning Score and Modified Early Warning Score scores

Table 6 shows the performance of NEWS and MEWS scores to predict outcomes with cut-off points. Excellent predictive performance of the NEWS score was found regarding mortality, with an AUC of 0.91 (95% CI: 0.88–0.94, *p*-value < 0.0001). The best cut-off value (>4) had a sensitivity of 71.87%, specificity of 95.5%, LR + of 15.98, LR- of 0.29, PPV of 57.5%, NPV of 97.6%, and 0.67% of Yuden index. The AUC values of the NEWS scores for ICU readmission, MV status, and multiple organ failure after readmission were considered fair. However, the poor predictive performance of the NEWS score was observed regarding the time to readmission and MV duration after readmission (Supplementary Figure 1).

**TABLE 6:** Receiver operating characteristic curve results of NEWS and MEWS scores to predicting outcomes.

	Outcomes	AUC (95% CI)	*p*-value	SN (95% CI)	SP (95% CI)	LR + (95% CI)	LR- (95% CI)	PPV (95% CI)	NPV (95% CI)	Youden Index	Cut-point
NEWS score	**Mortality** (Yes vs. No)	0.916 (0.885–0.941)	<0.0001*	71.87 (53.3–86.3)	95.50 (92.9–97.4)	15.98 (9.57–26.68)	0.29 (0.17–0.51)	57.5 (44.7–69.3)	97.6 (95.8–98.6)	0.673	>4
	**ICU Readmission** (Yes vs. No)	0.711 (0.665–0.755)	<0.0001*	88.00 (75.7–95.5)	50.00 (44.7–55.3)	1.76 (1.52–2.04)	0.24 (0.11–0.51)	19.7 (17.5–22.0)	96.8 (93.4–98.5)	0.380	>3
	**Discharge type** (Unplanned vs. Planned)	0.561 (0.512–0.610)	0.318	55.56 (30.8–78.5)	55.10 (50.0–60.1)	1.24 (0.81–1.90)	0.81 (0.48–1.36)	5.4 (3.6–8.0)	96.4 (94.1–97.8)	0.106	≤3
	**Time to readmission** (≥4 days vs. < 4 days)	0.676 (0.628–0.721)	<0.0001*	83.78 (68.0–93.8)	48.26 (43.1–53.5)	1.62 (1.36–1.92)	0.34 (0.16–0.70)	13.8 (11.9–16.0)	96.8 (93.5–98.4)	0.320	>3
	**MV Readmission** (Yes vs. No)	0.720 (0.674–0.763)	<0.0001*	87.18 (72.6–95.7)	48.79 (43.6–54.0)	1.70 (1.46–1.99)	0.26 (0.12–0.60)	15.2 (13.3 –17.3)	97.3 (94.1–98.8)	0.359	>3
	**Duration of MV readmission** (≥6 days vs. < 6 days)	0.675 (0.628–0.720)	0.0008*	81.82 (59.7–94.8)	46.91 (41.9–52.0)	1.54 (1.24–1.92)	0.39 (0.16–0.95)	8.1 (6.6–9.9)	97.8 (94.9–99.1)	0.287	>3
	**Multiple organ failure readmission** (Yes vs. No)	0.710 (0.664–0.754)	<0.0001*	87.50 (74.8–95.3)	49.72 (44.5–55.0)	1.74 (1.50–2.02)	0.25 (0.12–0.54)	18.7 (16.6–21.1)	96.8 (93.4–98.5)	0.372	>3
MEWS score	**Mortality** (Yes vs. No)	0.881 (0.846–0.911)	<0.0001*	68.75 (50.0–83.9)	98.94 (97.3–99.7)	64.97 (23.84–177.1)	0.32 (0.19–0.53)	84.6 (66.9–93.7)	97.4 (95.7–98.4)	0.676	>4
	**ICU Readmission** (Yes vs. No)	0.839 (0.799–0.873)	<0.0001*	80.00 (66.3–90.0)	88.61 (84.9–91.7)	7.02 (5.10–9.67)	0.23 (0.13–0.39)	49.4 (41.5–57.3)	97.0 (94.8–98.2)	0.686	>3
	**Discharge type** (Unplanned vs. Planned)	0.545 (0.495–0.593)	0.561	38.89 (17.3–64.3)	81.12 (76.9–84.9)	2.06 (1.11–3.81)	0.75 (0.52–1.09)	8.7 (4.9–14.9)	96.6 (95.2–97.7)	0.200	>3
	**Time to readmission** (≥4 days vs. <4 days)	0.826 (0.786–0.862)	<0.0001*	81.08 (64.8–92.0)	86.33 (82.4–89.6)	5.93 (4.40–7.99)	0.22 (0.11–0.43)	37.0 (30.3–44.2)	97.9 (95.9–98.9)	0.674	>3
	**MV Readmission** (Yes vs. No)	0.829 (0.789–0.864)	<0.0001*	79.49 (63.5–90.7)	86.52 (82.6–89.8)	5.90 (4.36–7.99)	0.24 (0.13–0.44)	38.2 (31.4–45.6)	97.6 (95.6–98.7)	0.660	>3
	**Duration of MV readmission** (≥6 days vs. <6 days)	0.813 (0.772–0.849)	<0.0001*	77.27 (54.6–92.2)	83.51 (79.4–87.1)	4.68 (3.41–6.44)	0.27 (0.13–0.59)	21.1 (16.3–26.9)	98.5 (96.7–99.3)	0.607	>3
	**Multiple organ failure readmission** (Yes vs. No)	0.833 (0.794–0.868)	<0.0001*	79.17 (65.0–89.5)	88.12 (84.3–91.3)	6.66 (4.86–9.14)	0.24 (0.14–0.41)	46.9 (39.2–54.8)	97.0 (94.8–98.2)	0.672	>3

LOS: Length of stay, MV: Mechanical ventilation, CI: Confidence interval, SN: Sensitivity; SP: Specificity; LR + : Positive likelihood ratio; LR-: Negative likelihood ratio; PPV: positive predictive value; NPV: Negative predictive value, *p-value < 0.05 considered significant.

According to the results, the MEWS score had a good predictive performance for all outcomes, except for the type of discharge, which was insignificant. Best performing predictive value of MEWS score was related to the mortality with AUC of 0.88 (95% CI: 0.84–0.91, *p*-value < 0.0001), and the best cut-off value (>4) had a value sensitivity of 68.75%, specificity of 98.94%, LR + of 64.97, LR- of 0.32, PPV of 84.6%, NPV of 97.4%, and 0.67% of Yuden index. The AUCs for predicting readmission, time to readmission, MV status, MV duration, and multiple organ failure varied between 0.81 and 0.83 (Supplementary Figure 2). The cut-off values for predicting readmission, time to readmission, MV status, MV duration, and multiple organ failure were three or more scores.

### Comparison of the outcome prediction ability between National Early Warning Score and Modified Early Warning Score

**FIGURE 1:**
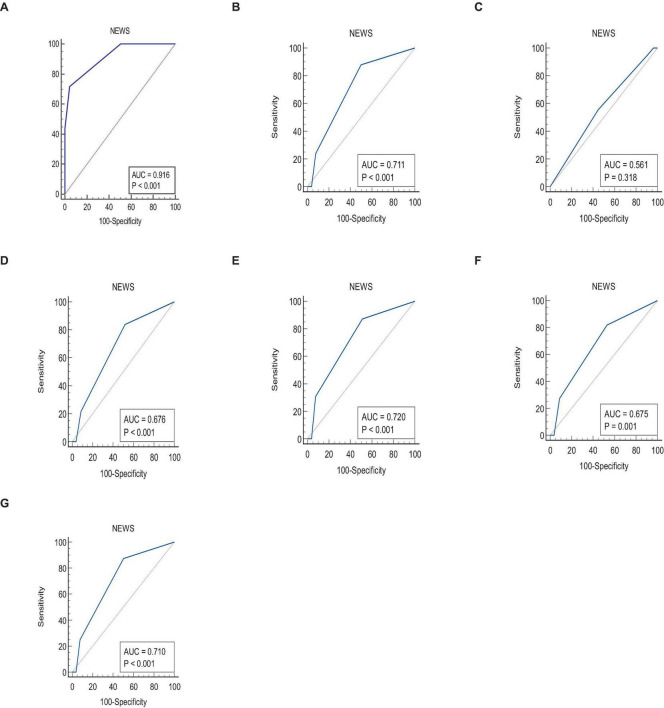
Comparison of ROC curves between NEWS and MEWS scores to predict **(A)** mortality, **(B)** ICU readmission, **(C)** unplanned discharge type, **(D)** time to readmission ≥ 4 days, **(E)** MV after readmission, **(F)** duration of MV ≥ 6 days, and **(G)** multiple organ failure after readmission.

A comparison of NEWS and MEWS AUCs was performed to predict the outcomes using the DeLong test, and the results are presented in Table 7. To predict mortality, the AUCs of NEWS and MEWS scores were 0.916 and 0.881, respectively, but this difference was not statistically significant (*p*-value = 0.082) (Figure 1A). However, the AUCs of the MEWS were significantly greater than NEWS for readmission (0.83 vs. 0.71, *p*-value < 0.0001) (Figure 1B), no significant difference for unplanned discharge types (Figure 1C), time to readmission (0.82 vs. 0.67, *p*-value < 0.0001) (Figure 1D), MV status, (0.82 vs. 0.72, *p*-value < 0.0001) (Figure 1E), MV duration (0.81 vs. 0.67, *p*-value < 0.0001) (Figure 1F), and multiple organ failure (0.83 vs. 0.71, *p*-value < 0.0001) (Figure 1G).

**TABLE 7:** Comparison of ROC curves between NEWS and MEWS scores to predict outcomes.

Outcomes	NEWS score			MEWS score			*P-value* *
		
	AUC	95% CI	*p*-value	AUC	95% CI	*p*-value	
Mortality	0.916	0.885–0.941	<0.0001	0.881	0.846–0.911	<0.0001	0.082
ICU Readmission	0.711	0.665–0.755	<0.0001	0.839	0.799–0.873	<0.0001	**<0.0001**
Discharge type	0.561	0.512–0.610	0.318	0.545	0.495–0.593	0.561	0.899
Time to readmission	0.676	0.628–0.721	<0.0001	0.826	0.786–0.862	<0.0001	**<0.0001**
MV Readmission	0.720	0.674–0.763	<0.0001	0.829	0.789–0.864	<0.0001	**<0.0001**
MV readmission duration	0.675	0.628–0.720	0.0008	0.813	0.772–0.849	<0.0001	**<0.0001**
Multiple organ failure	0.710	0.664–0.754	<0.0001	0.833	0.794–0.868	<0.0001	**<0.0001**

*P-value based on DeLong test to compare AUCs between NEWS and MEWS score for each outcome.

## Discussion

The MEWS and NEWS are relatively new scoring systems capable of predicting the prognosis of ICU patients. Few studies employ and compare the MEWS or NEWS as outcome predictors in ICU patients. In this multicenter, prospective, observational study, we compared the NEWS and MEWS scores to predict the outcomes in critically ill patients at the time of ICU discharge. The analysis from the multivariable logistic model showed that high MEWS and NEWS were the risk factors for readmission occurrences, time to readmission, mortality, MV status, MV duration, and multiple organ failure after readmission. By comparing these two scoring systems, we identified that there was no significant difference between the AUCs of the NEWS and the MEWS for predicting mortality (*P*-value = 0.082). In contrast, the prognostic accuracy of MEWS in other outcomes such as readmission occurrence, time to readmission, MV status, MV duration, and multiple organ failure excels the prognostic accuracy of NEWS score (*P*-value < 0.001). Such a result can be due to the fact that most problems that directly or indirectly affect the readmission of critically ill patients are related to respiratory dysfunction. The rate of readmission due to respiratory dysfunction in this study was almost 50%. In addition, our results show that male patients are more likely to be readmitted, have multiple organ failures, and have a longer MV duration. From a clinical perspective, these findings suggest that gender may also be an important consideration in discharge planning in addition to the use of NEWS and MEWS. However, further studies are needed to confirm this finding. Based on the findings of this study, we conclude that MEWS can be considered an effective prognostic tool for predicting all outcomes, and the NEWS score is a good predictor of mortality and ICU readmission in critically ill patients at the time of ICU discharge. Hence, we advocate for determining the MEWS and NEWS at ICU discharge as an assistive tool to make a better-informed decision.

Scoring systems can be used to measure the performance of one ICU over a time period, or used to compare the performance of different ICUs which allows ICUs to understand more about the quality of delivered care, audit themselves and assist them in decision-making, resource allocation, quality assessment programs and teaching. Each physician should consider that the decision regarding to whether the patients should or should not be admitted to the ICU is dependent on some other factors. These include the risk and complications of ICU admission/readmission, patients’ wishes, and the time lag when scores are calculated (usually 24 h after admission to the ICU), which means that clinical intervention may precede the calculation of the score. As the MEWS and NEWS scores includes all qSOFA variables, so they can serve as an accurate score in prediction of outcome even in patients with infection. Using a scores that includes a points-based risk score, such as the NEWS/MEWS, may improve teaching, the integration, and incorporation of early warning scores into clinical practice focused on identifying and managing patients at risk for poor outcome (30, 31). Our findings coincide with many similar studies. Consistent with this study, many previous studies have shown the NEWS and MEWS scores to be a decisive tool for the early identification of patients with a high risk of poor outcomes, including mortality and ICU readmission (32–34). Balshi et al. reported that the MEWS is associated with ICU readmission, and a score > 6 has an excellent accuracy as a prognostic predictor (32). A prospective observational study by Xie et al. showed good performance of MEWS for in-hospital mortality prediction, with AUC values at 0.83 in patients presenting to the emergency department (35). MEWS also helps predict the mortality of COVID-19 patients, with AUC values of 0.913 and 0.833 (36, 37). Lv et al. found that MEWS shows superiority over the quick Sequential Organ Function Assessment (qSOFA), Combination of Confusion, Urea, Respiratory Rate, Blood Pressure, and Age ≥ 65 (CURB-65), and NEWS scores in predicting hospital mortality, and NEWS showed superiority over the other scores in predicting ICU admission in patients with community-acquired pneumonia (CAP) (38). Klepstad et al. showed that the higher NEWS in gastrointestinal surgical patients at ICU discharge was the predictive factor of ICU readmission (33). Moreover, the study by Doðu et al. demonstrated that a NEWS value of >7.5 at the time of discharge from ICU estimates a high probability of ICU readmission within the first 48 h after discharge (34). However, in contrast to these findings, a study by Reini et al. showed that the MEWS at ICU discharge is not a predictor of ICU readmission (39). On the other hand, this finding might be influenced (as acknowledged by the authors) by the decision to withhold ICU readmission for 10 out of 15 patients discharged with a MEWS of 5 or more. MEWS and NEWS are widely used scoring systems in many countries, but differences between these studies, including study setting, population, and disease type, have led to differences in the predictive ability of these scoring systems.

The most important advantage of MEWS and NEWS scores compared with other scoring systems, such as APACHE IV, SOFA, and Simplified Acute Physiology Score (SAPS), are their simplicity. They consist of basic physiological measurements in contrast to APACHE IV, SOFA, and SAPS, which, for instance, need documentation of laboratory results, making them a simpler tool with facilitated assessment procedures (38, 39). The advantage of these simple scoring systems could be the early identification of patients who were becoming increasingly unstable. In addition, they could facilitate the discharge decision by the intensivist. Early identification of critically ill patients with poor outcomes at the time of discharge from the ICU can enable the appropriate allocation of limited resources, such as intensive care beds.

The strengths of this study were the multicenter prospective design with heterogeneous patients from the general ICUs of two hospitals, which adjusted the confounding variables and made the findings more generalizable. However, our study has several limitations. First, the patient selection criteria were inclusive (all patients aged 18 years and above admitted to general ICU); this creates a rather heterogeneous cohort, and due to the wide range of ICU admission causes, we could not see the reason for admission evaluated as a variable. Second, we have not presented the individual physiological parameters included in the MEWS and NEWS; identifying whether any of these parameters had a better predictive value than the others would be interesting. Third, we could not use multiple parametric models like MANOVA to adjust the potential correlation among outcomes due to the lack of normal distribution of outcomes as the pre-assumption required for multiple testing. Fourth, to deal with multiple outcomes, we considered mortality and ICU readmission as primary outcomes. However, secondary outcomes are then subsidiary, and the results concerning them can only have an exploratory rather than a confirmatory interpretation. In addition, the frequency of readmission in the ICU was low (50 from 410), so interpreting results related to secondary outcomes such as readmission time, MV readmission, and duration of MV readmission should be interpreted with caution. Nevertheless, due to the varied performance of MEWS and NEWS in other studies, future disease-specific studies are required to improve the accuracy and applicability of MEWS and NEWS.

## Conclusion

The MEWS and NEWS at the time of ICU discharge are independent predictors of ICU readmission and mortality. NEWS and MEWS scores greater than 4 have excellent and good accuracy in predicting mortality with 91 and 82% AUCs, respectively. In addition, scores greater than 3 have good and fair accuracy in predicting ICU readmission with AUCs of 83 and 71%, respectively. We found that the MEWS showed superiority over the NEWS score in predicting ICU readmission, time to readmission, MV readmission, MV duration, and multiple organ failure.

## Data availability statement

The raw data supporting the conclusions of this article will be made available by the authors, without undue reservation.

## Ethics statement

The studies involving human participants were reviewed and approved by the Ethics Committees of Islamic Azad University-Tabriz Branch (IR.TBZMED.REC.1397.994). The patients/participants provided their written informed consent to participate in this study.

## Author contributions

FR-B, AM, SS, NS, and AV-A: study concept and design. SHS, ZO, M-SH, and SS: analysis and interpretation of data. SHS and AS: acquisition of data and drafting of the manuscript. FR-B, ZO, and SS: critical revision of the manuscript for important intellectual content. FR-B and AM: statistical analysis. All authors contributed to the article and approved the submitted version.

## Footnotes

1
https://www.medcalc.org/calc/diagnostic_test.php
