# Supplementary material for: Prognostic value of National Early Warning Score and Modified Early Warning Score on intensive care unit readmission and mortality: A prospective observational study
Source: Front Med (Lausanne). 2022 Aug 4;9:938005. doi: 10.3389/fmed.2022.938005 (PMC9386480; doi:10.3389/fmed.2022.938005)
Supplement: Supplementary file 1 [file Data_Sheet_1.pdf]

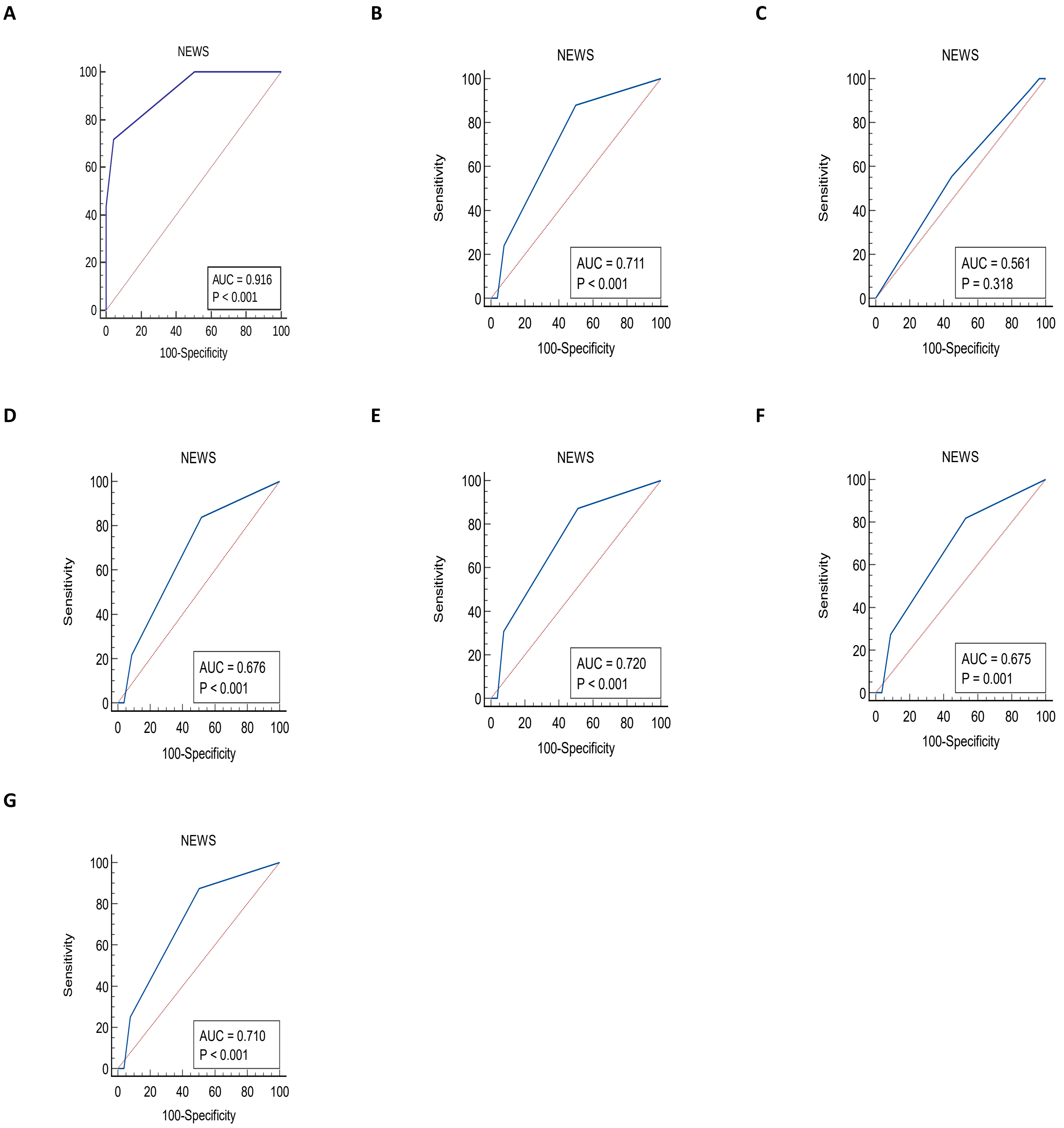

Supplementary Figure 1: ROC curves of NEWS score to predict (A) mortality, (B) ICU readmission, (C) unplanned discharge type, (D) interval of readmission ≥ 4 days, (E) MV readmission, (F) duration of MV ≥6 days, and (G) multiple organ failure

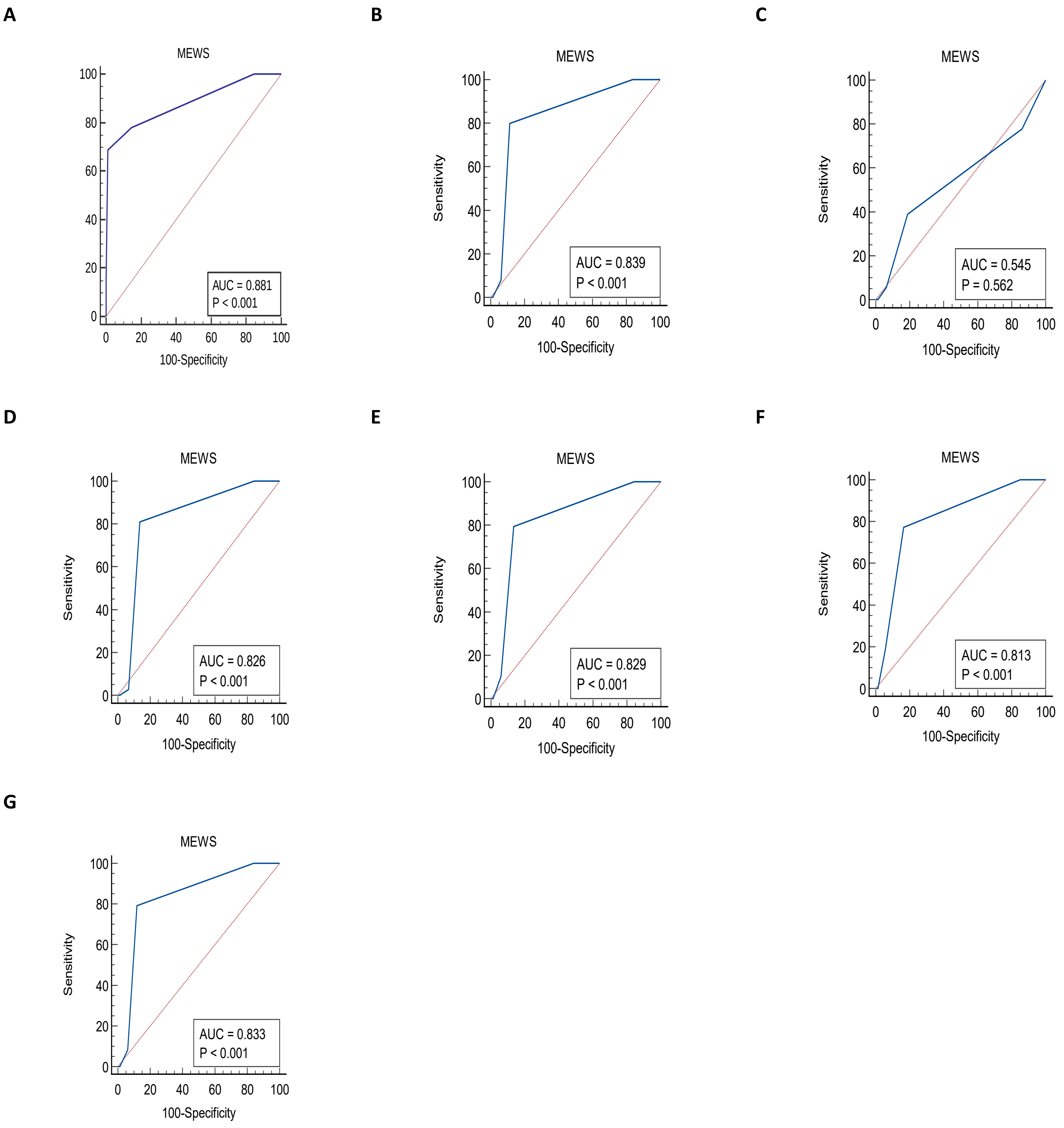
A
MEWS
100-Specificity
AUC = 0.881
P < 0.001
B
MEWS
Sensitivity
100-Specificity
AUC = 0.839
P < 0.001
C
MEWS
Sensitivity
100-Specificity
AUC = 0.545
P = 0.562
D
MEWS
Sensitivity
100-Specificity
AUC = 0.826
P < 0.001
E
MEWS
Sensitivity
100-Specificity
AUC = 0.829
P < 0.001
F
MEWS
Sensitivity
100-Specificity
AUC = 0.813
P < 0.001
G
MEWS
Sensitivity
100-Specificity
AUC = 0.833
P < 0.001

Supplementary Figure 2: ROC curves of MEWS score to predict (A) mortality, (B) ICU readmission, (C) unplanned discharge type, (D) interval of readmission ≥ 4 days, (E) MV readmission, (F) duration of MV ≥6 days, and (G) multiple organ failure
